# Luteolin prevents cadmium-induced PC12 cell death by suppressing the Akt/mTOR signaling pathway

**DOI:** 10.1097/MD.0000000000040372

**Published:** 2024-11-01

**Authors:** Xuan Zhang, Wenjie Xu, Huan Li, Dapeng Ruan, Siyuan Chen, Na Chu, Quan Zhen, Yun Wang

**Affiliations:** a School of Public Health, Bengbu Medical College, Bengbu, Anhui, China; b Anhui Provincial Center for Disease Control and Prevention, Hefei, Anhui, China.

**Keywords:** cadmium, cell apoptosis, luteolin, protein kinase B (Akt)/mammalian target of rapamycin (mTOR) signaling pathway

## Abstract

Cadmium (Cd) is an environmental pollutant that can cause neurodegenerative disorders. Luteolin (Lut) is a natural flavonoid compound. However, whether Lut protects against Cd-induced nerve cell death remains unclear. In the present study, PC12 cells were used to investigate the neuroprotective effect of Lut against Cd poisoning. Changes in cell viability, apoptosis, B-cell lymphoma-2 (Bcl-2) and Bcl-2-associated X protein expression, and protein kinase B (Akt)/mammalian target of rapamycin (mTOR) pathway activity were analyzed by the 3-(4,5-dimethylthiazol-2-yl)-2,5-diphenyltetrazolium bromide assay, Hoechst 33258 staining, flow cytometry, and western blotting. Lut markedly attenuated the Cd-induced reduction in cell viability, nuclear fragmentation, condensation, and the decrease in the Bcl-2/Bcl-2-associated X protein ratio in PC12 cells. Furthermore, Lut blocked the Cd-mediated activation of the Akt/mTOR signaling pathway. Moreover, inhibition of the Akt/mTOR signaling pathway with LY294002 (a PI3K inhibitor) enhanced the protective effect of Lut against Cd-induced cell death by suppressing Cd-induced activation of Akt, mTOR, and eukaryotic initiation factor 4E binding protein 1. The results showed that Lut prevented Cd-induced cell death partly by blocking the Akt/mTOR signaling pathway. Lut may be a potential agent for preventing Cd-induced nerve cell damage and neurodegenerative diseases.

## 1. Introduction

Cadmium (Cd) is one of the most dangerous heavy metal pollutants, and its primary source is industrial waste and agricultural and urban sewage discharge. It has a long biological half-life (15–20 years) and can accumulate substantially in the human body; additionally, it has toxic effects on multiple organs and systems.^[1]^ Clinical and epidemiological data have shown that Cd exposure is associated with various diseases, including renal tubular dysfunction, osteoporosis, cardiovascular diseases, and cognitive impairment.^[2–4]^ In addition, numerous studies have demonstrated that Cd poisoning may be an etiological factor in neurodegenerative diseases, such as Parkinson disease and Alzheimer disease.^[5]^ Thus, identifying a novel therapeutic target and strategy to prevent Cd neurotoxicity is essential. To this end, many attempts have been made, such as using natural products to prevent and treat neurodegenerative diseases.^[6]^

The protein kinase B (Akt)/mammalian target of rapamycin (mTOR) signaling pathway is downstream of PI3K and plays a vital role in regulating cell proliferation, apoptosis, and metabolism.^[7]^ mTOR activity is altered in various nervous system diseases, including brain tumors, tuberous sclerosis, cortical dysplasias, and neurodegenerative disorders such as Alzheimer disease, Parkinson disease, and Huntington disease.^[8]^ Therefore, inhibiting mTOR activity may reduce the neurodegenerative changes associated with these diseases. Several studies have demonstrated that Cd induces neuronal apoptosis by activating the Akt/mTOR signaling pathway; blocking the activation of the Akt/mTOR signaling pathway can markedly attenuate Cd-induced brain damage and neuronal death in mice.^[5,9,10]^

**Figure 1.**
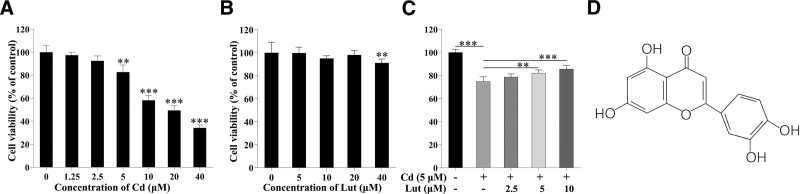
Lut attenuates the Cd-induced decrease in viability in PC12 cells. (A) PC12 cells were treated with different concentrations of Cd for 24 h. (B) PC12 cells were treated with 5–40 μM Lut for 24 h. (C) PC12 cells were exposed to 5 μM Cd with or without 2.5, 5, or 10 μM Lut for 24 h. (D) Chemical structure of Lut. The cell viability data are reported as the mean ± SD (n = 6). **P < .01, ***P < .001, Lut = luteolin, Cd = cadmium.

Luteolin (Lut, the chemical structure is shown in Figure 1D), which is a natural flavonoid compound first isolated from the branches, stems, and leaves of *Reseda odorata* L., has been found in fruits, vegetables, and herbs such as celery, carrots, broccoli, and honeysuckle.^[11–13]^ In vivo and in vitro studies have confirmed the protective effects of Lut against oxidative stress and apoptosis induced by various toxicants in multiple organs and cell lines.^[14–16]^ Moreover, since Lut can cross the blood–brain barrier, it is suitable for treating central nervous system diseases such as neurodegenerative disease.^[17,18]^ Some studies suggest that Lut can ameliorate inorganic mercury-induced cardiac histopathological damage in Wistar rats, improve the behavior of Ts65Dn mice with Down syndrome, promote hippocampal neurogenesis, protect neurons from microglial-mediated LPS neurotoxicity, attenuate neuronal damage induced by PbAc, and protect against cobalt chloride-induced behavioral, morphological and neurochemical alterations in Wistar rats.^[19–23]^ These studies show that Lut has specific neuroprotective effects. Therefore, we hypothesize that Lut can prevent or treat Cd-induced nerve cell damage.

In the present study, we investigated the protective effects of Lut against Cd-induced damage in PC12 cells. Highly differentiated PC12 cells are similar to neurons in the cortical layer of the brain, and they have the differentiation potential and properties of sympathetic neurons.

## 2. Materials and methods

### 2.1. Cells and reagents

Rat pheochromocytoma cells (highly differentiated PC12 cells) were purchased from Guangzhou Cellcook Biotech Co., Ltd (China). RPMI-1640 medium was purchased from Thermo Fisher (USA). Fetal bovine serum was purchased from Clark (USA). Lut and Cadmium chloride (CdCl_2_) were purchased from Sigma Aldrich (USA). The 3-(4,5-dimethylthiazol-2-yl)-2,5-diphenyltetrazolium bromide (MTT) solution (0.5%) and DMSO were purchased from Solarbio (China). A Hoechst staining kit and annexin V-fluorescein isothiocyanate (FITC) apoptosis detection kit were purchased from Beyotime (China). Primary monoclonal antibodies against the following proteins were obtained from Cell Signaling Technology (USA): Akt, p-Akt, mTOR, and p-mTOR. Primary antibodies against the following proteins were purchased from Proteintech Group (China): B-cell lymphoma-2 (Bcl-2), Bcl-2-associated X protein (Bax), and β-actin. HRP goat anti-rabbit IgG was obtained from Cell Signaling Technology (USA). HRP goat anti-mouse IgG was purchased from Biosharp (China). Cell lysis buffer for western blotting and IP, PMSF, a BCA protein assay kit, and a hypersensitive ECL chemiluminescence kit were purchased from Beyotime (China).

### 2.2. Cell culture

PC12 cells were cultured in RPMI-1640 medium supplemented with 10% fetal bovine serum, 100 U/mL penicillin, and 0.1 mg/mL streptomycin in 5% CO_2_ and 95% air at 37 °C. The PC12 cells were mycoplasma negative.

### 2.3. MTT assay for cell viability

PC12 cells (3 × 10^4^/mL) were seeded in 96-well plates and cultured overnight. Then, the cells were treated with different concentrations of Cd (1.25, 2.5, 5, 10, 20, and 40 μM) or Lut (5, 10, 20, and 40 μM) for 24 hours or treated with Cd (5 μM) alone or in combination with Lut at 2.5, 5, and 10 μM; there were 6 replicates for each concentration. Next, the cells were incubated with 10 μL MTT solution (5 mg/mL) for 4 hours. The absorbance (A) was measured at a wavelength of 490 nm using a microplate reader. The relative survival rate in each group was calculated as follows: $Cell\;relative\;survival\;rate=\frac{experimental\;group\;\left(A\;value\right)}{control\;group\;\left(A\;value\right)}\times 100\%$.^[24]^ All the experiments were repeated 3 times.

### 2.4. Hoechst 33258 staining of apoptotic cells

PC12 cells in the logarithmic growth phase were seeded at a density of 2 × 10^6^ cells/well in 6-well plates, cultured overnight, and treated with Cd (5 μM) with or without various concentrations of Lut (0, 2.5, 5, and 10 μM) for 24 hours. According to the manufacturer’s instructions, apoptosis-related markers (such as nuclear chromatin) in PC12 cells were examined by Hoechst 33258 staining. Nuclear chromatin in each group was observed under an inverted fluorescence microscope at 400×, and photographs were taken.

### 2.5. Analysis of apoptosis by flow cytometry

According to the manufacturer’s protocol, experiments were performed using an annexin V-FITC apoptosis detection kit (BD, Biosciences, Franklin Lakes, NJ). Briefly, the cells were harvested, washed, and stained with annexin-FITC (1 μL) and propidium iodide (PI 5 μL) double staining buffer at room temperature for 20 minutes. The percentage of apoptosis was measured by a BD-FACSVerse flow cytometer (BD Biosciences). The experiment was repeated 3 times. The percentages of early apoptotic (Annexin V^+^/PI^‐^) and late apoptotic (annexin V^+^/PI^+^) cells were determined.

### 2.6. Western blot analysis

PC12 cells in the logarithmic growth phase were collected and lysed with cell lysis buffer. The protein concentration was measured by the BCA assay. Next, 40 μg total protein was loaded in each lane, and the proteins were separated by 10% SDS–PAGE and transferred to PVDF membranes. After being blocked in blocking solution for 2 hours at room temperature, the membranes were incubated with primary antibodies against Bcl-2 (1:1000), Bax (1:10,000), Akt (1:1000), p-Akt (1:1000), mTOR (1:1000), p-mTOR (1:1000), eukaryotic initiation factor 4E binding protein 1 [4E-BP-1] (1:1000), *P*-4E-BP-1 (1:1000), or β-actin(1:20,000) overnight at 4 °C. After being washed, the membranes were incubated with HRP-conjugated goat anti-rabbit IgG or goat anti-mouse IgG secondary antibodies (1:2000) for 1 hour at room temperature. Finally, images were obtained after ECL development, and the gray values of the bands were analyzed using NIH ImageJ software. The experiment was repeated 3 times.

### 2.7. Statistical analysis

SPSS 25.0 software was used (IBM Corp., Armonk, NY) to analyze the data, and the data are presented as the mean ± standard deviation. Graphs were made with GraphPad Prism 8.0. Student *t* test was used to analyze differences between 2 groups, and multiple comparisons were performed by one-way analysis of variance followed by Dunnett test. *P* < .05 indicates a significant difference.

### 2.8. Ethics approval statement

Because the experiment does not involve animals and clinical research, this study does not need to be approved by moral and ethical clerks.

## 3. Results

### 3.1. Lut attenuates the Cd-induced reduction in PC12 cell viability and morphological alterations

To determine the appropriate concentration of Cd for use in the experiments, we assessed the viability of PC12 cells after treatment with Cd. As shown in Figure 1A, cell viability decreased in a dose-dependent manner after treatment with Cd. The effect of Lut on the viability of PC12 cells was subsequently investigated. As shown in Figure 1B, treatment with Lut at concentrations up to 20 μM did not significantly affect cell viability, indicating that at concentrations of 0 to 10 μM, Lut was not toxic to PC12 cells; therefore, we evaluated the protective effect of these concentrations Lut against cadmium (Cd)-induced cytotoxicity in PC12 cells.

We treated PC12 cells with Cd (5 μM) alone or in combination with Lut (2.5, 5, or 10 μM) for 24 hours to test the protective effect of Lut against Cd-induced neuronal cell death. The viability of PC12 cells was significantly lower in the Cd (5 μM) group than in the other groups. Compared with the control group, the Cd + Lut5 and Cd + Lut10 groups exhibited decreased survival rates. However, the Cd + Lut5 and Cd + Lut10 groups had higher survival rates than the Cd-treated group, which was not administered Lut (Fig. 1C). These results suggest that Lut can prevent Cd-induced PC12 cell death.

### 3.2. Lut prevents Cd-induced apoptosis in PC12 cells

**Figure 2.**
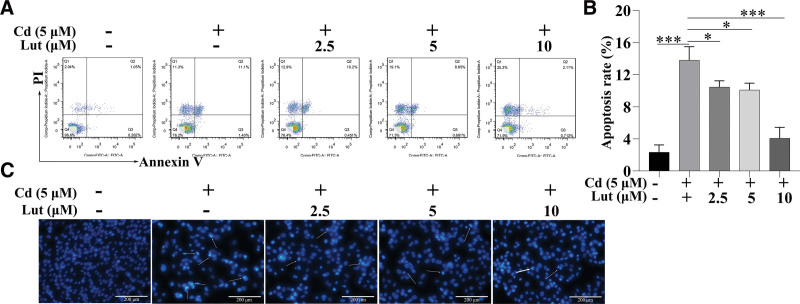
Lut prevented Cd-induced apoptosis in PC12 cells. (A, B) Apoptosis in PC12 cells was measured by flow cytometry. A representative experimental result is shown. (C) The morphological characteristics of PC12 cells exposed to 5 μM Cd with or without 2.5, 5, or 10 μM Lut for 24 h were determined by Hoechst 33258 staining and fluorescence microscopy. The arrows indicates apoptotic cells with nuclear condensation. The original magnification is 400×. Scale bar: 200 μm. The data represent the mean values of 3 independent experiments. *P < .05, ***P < .001, Lut = luteolin, Cd = cadmium, SD = standard deviation.

We treated PC12 cells with Cd (5 μM) alone or in combination with Lut (2.5, 5, or 10 μM) for 24 hours to evaluate the effect of Lut on Cd-induced apoptosis. Figure 2A and B shows the apoptosis data and the results of the data analysis, respectively. Q2 represents cells in late apoptosis, and Q3 represents cells in early apoptosis. Compared with that in the control group, the apoptosis rate in the Cd treatment group was significantly increased. Compared with that in the Cd group, the apoptosis rate in the Cd + Lut2.5, Cd + Lut5, and Cd + Lut10 groups was markedly decreased (Fig. 2B). Then, we used Hoechst 33258 staining to assess nuclear fragmentation and condensation, which are hallmarks of apoptosis.^[25]^ We found that treatment with Cd (5 μM) for 24 hours significantly increased nuclear fragmentation and condensation, and these effects were markedly attenuated by Lut (Fig. 2C). These results revealed that Lut can protect against Cd-induced PC12 cell apoptosis.

**Figure 3.**
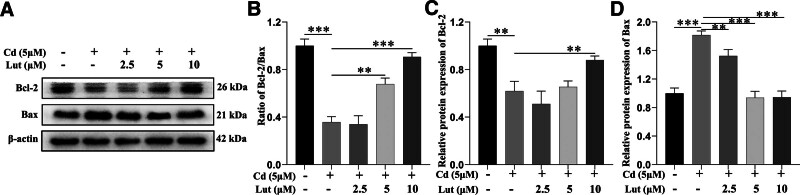
Effect of Lut on the expression of Bcl-2 and Bax in Cd-induced PC12 cells. PC12 cells were treated with 5 μM Cd and 2.5, 5, or 10 μM Lut for 24 h. (A) The protein levels of Bcl-2, Bax, and β-actin in PC12 cells were examined by immunoblotting. (B) The relative ratio of Bcl-2/Bax is shown. (C, D) The band intensities of Bcl-2 and Bax were normalized to the expression levels of β-actin. The data represent the mean values of 3 independent experiments. **P < .01, ***P < .001, Lut = luteolin, Cd = cadmium, Bcl-2 = B-cell lymphoma-2, Bax = Bcl-2-associated X protein.

To gain more insight into the mechanism by which Lut exerts neuroprotective effects by reversing Cd-induced apoptosis, we assessed the protein levels of Bcl-2 and Bax in PC12 cells. The western blot results showed that, compared with the control treatment, treatment with Cd significantly decreased the Bcl-2/Bax ratio in PC12 cells. Lut treatment significantly alleviated this decrease (Fig. 3).

### 3.3. Lut inhibits Cd-induced PC12 cell death by blocking the Akt/mTOR signaling pathway

**Figure 4.**
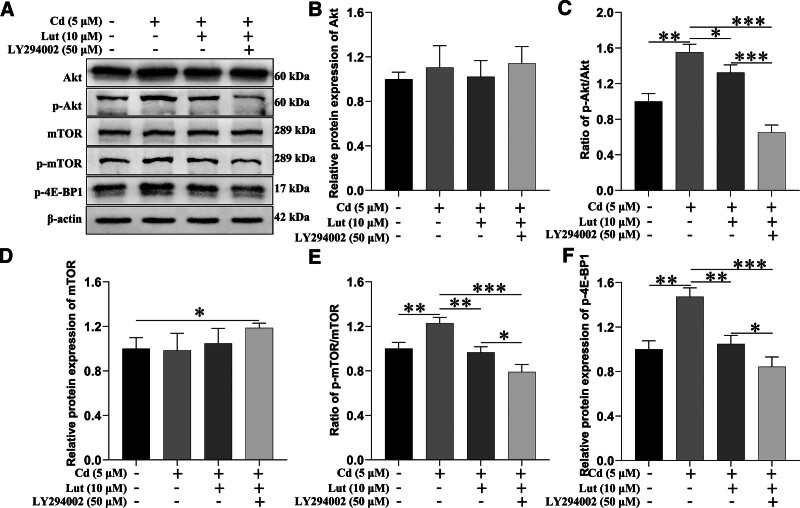
Lut alleviated Cd-induced PC12 cell death by blocking the Akt/mTOR signaling pathway. PC12 cells were pretreated with or without LY294002 (50 μM) for 2 h and then exposed to Cd (5 μM) with or without Lut (10 μM) for 24 h. (A) The protein levels of Akt, p-Akt, mTOR, p-mTOR, *P*-4E-BP1, and β-actin in PC12 cells were examined by immunoblotting. (B, D, F) The Akt, mTOR, and *P*-4E-BP-1 band intensities were normalized to the expression levels of β-actin. (C, E) The relative ratios of p-Akt/Akt and p-mTOR/mTOR are shown. The data represent the mean values of 3 independent experiments. *P < .05, **P < .01, ***P < .001, Lut = luteolin, Cd = cadmium, Akt = protein kinase B, p-Akt = phosphorylated protein kinase B, mTOR = mammalian target of rapamycin, p-mTOR = phosphorylated mammalian target of rapamycin, *P*-4E-BP-1 = phosphorylated eukaryotic initiation factor 4E binding protein 1.

The Akt/mTOR signaling pathway is crucial for controlling cell growth, maintaining cell viability, and determining a cell’s life span.^[26]^ Therefore, we hypothesized that Lut could prevent Cd-induced PC12 cell apoptosis by inhibiting the Akt/mTOR pathway. To test this hypothesis, PC12 cells were pretreated with or without LY294002 (2-(4-morpholinyl)-8-phenyl-4H-1-benzopyran-4-one, a PI3K inhibitor, 50 μM) for 2 hours and then exposed to Cd (5 μM) and Lut (10 μM) for 24 hours, after which western blot analysis was performed. We found that Lut attenuated Cd-induced Akt, mTOR, and 4E-BP1 phosphorylation. Furthermore, treatment with Lut plus LY294002 had a more substantial inhibitory effect on the Cd-induced activation of Akt, mTOR, and 4E-BP-1 (Fig. 4).

**Figure 5.**
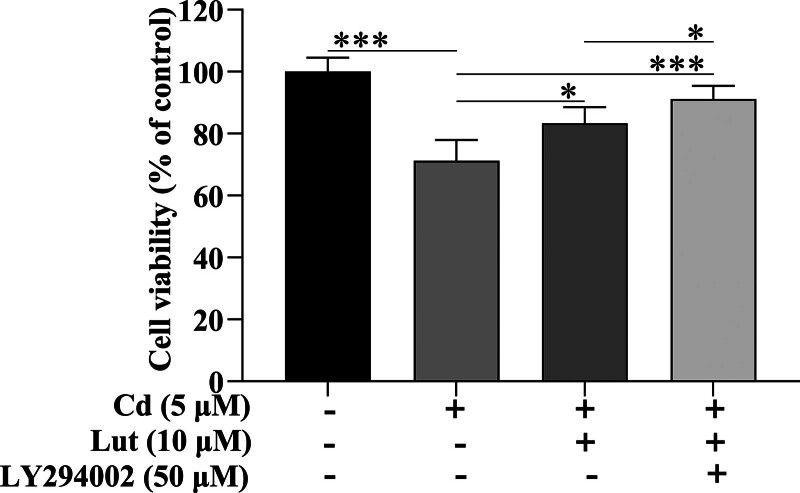
Effects of Lut and LY294002 on the viability of Cd-induced PC12 cells. PC12 cells were pretreated with or without LY294002 (50 μM) for 2 h and then exposed to Cd (5 μM) with or without Lut (10 μM) for 24 h. Cell viability was evaluated by the MTT assay. The results are reported as the mean ± SD (n = 6). *P < .05, **P < .01, ***P < .001, Lut = luteolin, Cd = cadmium, MTT = 3-(4,5-dimethylazol-2-yl)-2,5-diphenyl tetrazolium bromide, SD = standard deviation.

Consistent with this finding, treatment with Lut and LY294002 also inhibited Cd-induced cell death more strongly than Lut alone (Fig. 5). Our data suggest that Lut can partially prevent Cd-induced PC12 cell death by blocking the Akt/mTOR pathway.

## 4. Discussion

Cadmium is a heavy metal and a highly toxic pollutant that is prevalent in the environment due to rapid industrial development and the advancement of modern technologies.^[27]^ Previous studies have shown that acute and chronic cadmium exposure can lead to the death of various cell types.^[28–30]^ Therefore, taking active measures to prevent Cd poisoning is of clinical value, and this conclusion is supported by scientific evidence. Lut is a natural flavonoid compound in foods derived from plants.^[11]^ It can inhibit the toxicity of many kinds of heavy metals on HL7702 hepatocytes.^[31]^ Here, we present evidence for the first time that Lut protects against Cd-induced PC12 cell death. Lut increases the viability of PC12 cells exposed to Cd. Our findings suggest that Lut may be a potent neuroprotective phytochemical present in foods derived from plants.

Cell death is the final stage of a cell’s life and can be caused by exogenous or endogenous substances. Apoptosis is a well-established form of regulated cell death. As a specific suicide program, apoptosis is a type of cell death that occurs in an ordered, regulated fashion. Several investigators have reported that plant extracts, such as beta-carotene, curcumin, astilbin, alpha-mangostin, and celastrol, inhibit Cd-mediated oxidative stress-related apoptosis.^[10,32–35]^ In the present study, we found that Lut markedly suppressed Cd-induced apoptosis and strongly blocked the nuclear fragmentation and condensation induced by Cd exposure; Lut also suppressed the Cd-induced decrease in the Bcl-2/Bax ratio. These results are consistent with recent studies on the ability of natural compounds to inhibit Cd toxicity. Our data suggest that Lut can attenuate Cd-induced cell death by blocking apoptosis.

Cd exposure can activate the Akt/mTOR signaling pathway, leading to apoptosis in a variety of cells.^[9,36–38]^ Liu Y et al treated cells with Lut and reported that it significantly inhibited methylglyoxal-induced activation of the mTOR/4E-BP-1 signaling pathway and reduced apoptosis in PC12 cells.^[39]^ However, whether the inhibitory effect of Lut on Cd-induced apoptosis in PC12 cells is associated with inhibition of the Akt/mTOR signaling pathway has not been determined. To further investigate the involvement of this pathway in the effect of Lut, we treated PC12 cells with Cd alone or in combination with Lut. Our results showed that Lut inhibited Cd-induced Akt and mTOR phosphorylation in PC12 cells. In a similar manner, celastrol was found to prevent Cd-induced neuronal apoptosis by inhibiting the Akt-mediated mTOR pathway.^[40]^

Additionally, several PI3K/Akt/mTOR inhibitors, such as rapamycin, LY294002, and wortmannin, have been shown to block Cd-induced phosphorylation of mTOR and 4E-BP1 and markedly inhibit Cd-induced apoptosis.^[9,10,37]^ Consequently, we hypothesized that the inhibitory effect of Lut could be promoted by blocking this pathway. The present study supports these previous finding; our results showed that treatment with Lut and LY294002 more significantly inhibited the Cd-induced phosphorylation of Akt, mTOR, and 4E-BP-1 than treatment with Lut alone. Consistent with these findings, treatment with Lut and LY294002 also rescued cells from Cd-induced death more strongly than treatment with Lut alone.

In summary, Cd can reduce PC12 cell viability and promote apoptosis in PC12 cells, and Lut can attenuate Cd-induced PC12 cell damage by suppressing the Akt/mTOR pathway. The results of this study could lead to the use of Lut to prevent or treat Cd-induced neurotoxicity in nerve cells.

## Author contributions

**Data curation:** Xuan Zhang, Huan Li, Dapeng Ruan, Siyuan Chen, Na Chu.

**Formal analysis:** Xuan Zhang.

**Investigation:** Xuan Zhang, Wenjie Xu, Yun Wang.

**Project administration:** Yun Wang.

**Writing – original draft:** Xuan Zhang.

**Writing – review & editing:** Quan Zhen.

## Correction

This article was originally published without funding and support information. Funding Information (grant numbers 2022AH051454 and 2021BYZD030 were added to the funding information for the Natural Science Foundation of Anhui Universities and the Natural Science Foundation of Bengbu Medical College respectively) and Support Information (projects funded by grant numbers BYYCXZ21044 and BYYCX22066 were added to the support information for the Bengbu Medical College) have now been added in the online version.
